# Urticaria and angioedema

**DOI:** 10.1186/s13223-018-0288-z

**Published:** 2018-09-12

**Authors:** Amin Kanani, Stephen D. Betschel, Richard Warrington

**Affiliations:** 1 Division of Allergy and Immunology, Department of Medicine, University of British Columbia, Vancouver, BC Canada; 2 Division of Allergy and Immunology, Department of Medicine, St. Michael’s Hospital, University of Toronto, Toronto, ON Canada; 3 Section of Allergy & Clinical Immunology, Department of Internal Medicine, University of Manitoba, Winnipeg, MB Canada

**Keywords:** Urticaria, Angioedema, Acute urticaria, Chronic urticaria, Chronic spontaneous urticaria, Inducible urticaria, Hereditary angioedema, Acquired angioedema

## Abstract

Urticaria (hives) is a common disorder that often presents with angioedema (swelling that occurs beneath the skin). It is generally classified as acute or chronic. Second-generation, non-sedating, non-impairing histamine type 1 (H1)-receptor antihistamines represent the mainstay of therapy for both acute and chronic urticaria. Angioedema can occur in the absence of urticaria and can be broadly divided into histamine-mediated and non-histamine-mediated angioedema. Histamine-mediated angioedema can be allergic, pseudoallergic or idiopathic. Non-histamine mediated angioedema is largely driven by bradykinin and can be hereditary, acquired or drug-induced, such as with angiotensin-converting enzyme inhibitors. Although bradykinin-mediated angioedema is often self-limited, laryngeal involvement can lead to fatal asphyxiation. The mainstay of management for angioedema is to avoid specific triggers, if possible. For hereditary angioedema, there are specifically licensed treatments that can be used for the management of acute attacks, or for prophylaxis in order to prevent attacks. In this article, the authors will review the causes, diagnosis and management of urticaria (with or without angioedema) and isolated angioedema. The diagnostic and therapeutic approaches to these two conditions are considerably different, and this review is designed to highlight these differences to the reader.

## Background

**Fig. 1:**
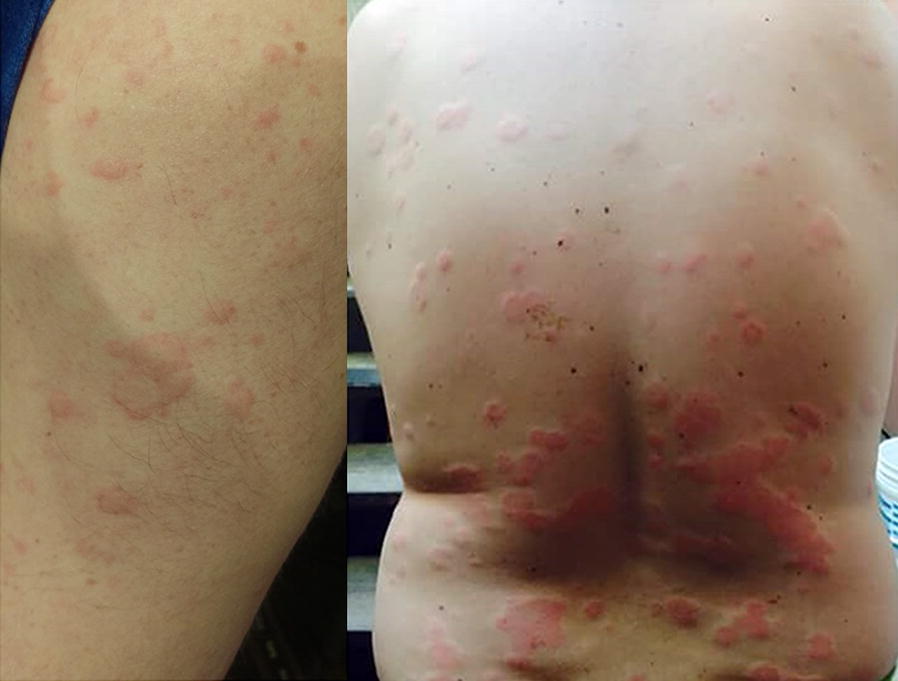
**Urticaria (hives)**

Urticaria is a common disorder, occurring in 15–25% of individuals at some point in life [1, 2]. It is characterized by recurrent, pruritic, wheals with pale, central swelling and surrounding epidermal erythema which can appear over any part of the body (see Fig. 1). The lesions can range in size from a few millimeters to several centimeters in diameter, and are often transient, resolving within about 24 h without scarring; however, some lesions may last up to 48 h [1–4]. Approximately 40% of patients with urticaria also experience angioedema (swelling that occurs beneath the skin) [3].

Mast cells are the primary effector cells in urticaria and in many cases of angioedema. These cells are widely distributed in the skin, mucosa, and other areas of the body, and have high-affinity immunoglobulin E (IgE) receptors. Mast cell degranulation leads to the rapid release of various inflammatory mediators, such as histamine, leukotrienes and prostaglandins, which, in turn, cause vasodilation and leakage of plasma in and below the skin. There is also a more delayed (4–8 h) secretion of inflammatory cytokines (e.g., tumor necrosis factor, interleukin 4 and 5) that potentially leads to further inflammatory responses and longer-lasting lesions [1].

**Fig. 2:**
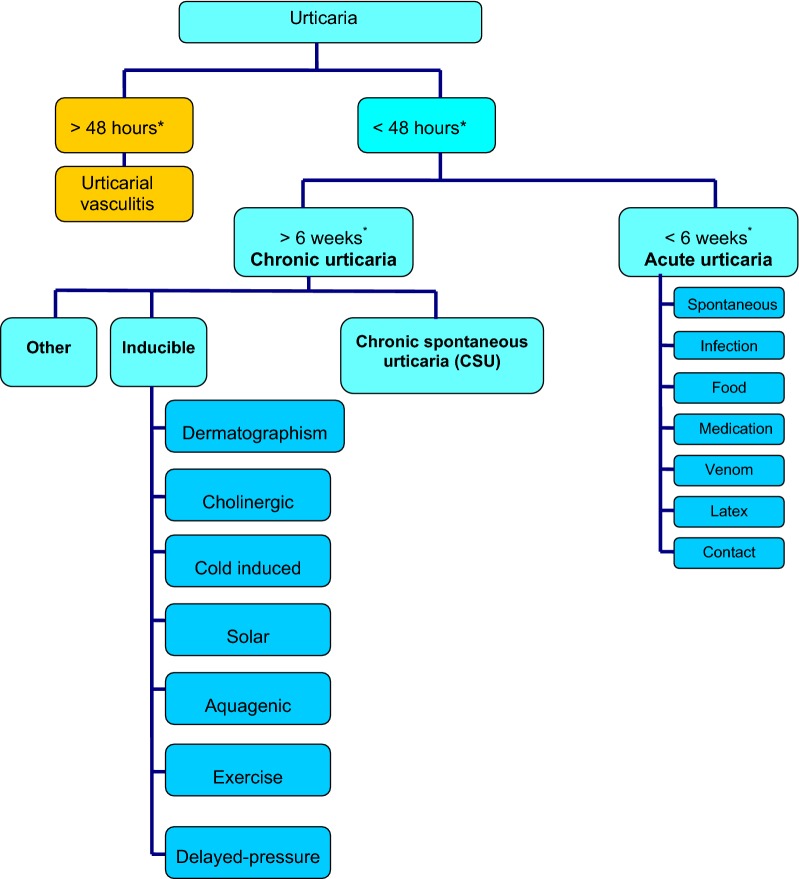
**Classification of urticaria: overview.** *The 48-h cut-off refers to individual lesions, while the 6-week cut-off refers to the condition as a whole

Urticaria is generally classified as acute or chronic, depending on the duration of symptoms and the presence or absence of inducing stimuli (see Fig. 2) [5–7]. Acute urticaria refers to urticaria with or without angioedema which lasts less than 6 weeks. Chronic urticaria is defined as urticaria with or without angioedema that has been continuous or intermittent for at least 6 weeks. Chronic urticaria can be further classified as chronic spontaneous urticaria (CSU) and inducible urticaria. The latter represents a distinct subgroup of chronic urticaria that is induced by physical stimuli, such as scratching (dermatographism, a common form of physical urticaria), cold, heat, sunlight, vibration and pressure.

Although acute urticaria can generally be easily managed and is associated with a good prognosis, chronic, severe urticaria is often associated with significant morbidity and diminished quality of life (QOL) [8]. Inducible urticaria also tends to be more severe and long-lasting, and can sometimes be challenging to treat [1, 3].

The first section of this article will focus on the causes, diagnosis and management of the most common types of urticaria (with or without angioedema). The second section will review the work-up and management of angioedema without urticaria. This type of angioedema can be broadly divided into histamine-mediated and non-histamine-mediated angioedema. Distinguishing between these two types of angioedema is important as investigations and management differ considerably.

## Urticaria

### Classification and etiology

#### Chronic urticaria

The prevalence of chronic urticaria has been estimated to be 0.5–5%. Chronic urticaria is more common in adults, with a peak age of onset between 20 and 40 years, and it affects women more frequently than men [3, 5–7]. In CSU, an external trigger cannot usually be identified (see Fig. 2). In approximately 45% of these patients, circulating immunoglobulin G (IgG) autoantibodies recognize IgE antibodies or the alpha subunit of the high-affinity IgE receptor on dermal mast cells and basophils, leading to chronic stimulation of these cells and the release of histamine and other inflammatory mediators that cause urticaria and angioedema. CSU is also associated with antithyroid antibodies in approximately 27% of cases [5–7]. Numerous other autoimmune disorders, including rheumatoid arthritis, systemic lupus erythematosus (SLE), dermatomyositis, polymyositis, Sjogren’s syndrome, and Still’s disease, have been associated with chronic urticaria. Investigations for these conditions are not warranted unless there are clear features of their presence on clinical evaluation. A variety of chronic infections have also been reported to be associated with chronic urticaria, including viral hepatitis B and C, Epstein-Barr virus, herpes simplex virus, *Helicobacter pylori* infections, and helminth parasitic infections. With the exception of parasitic infections, these are not believed to play a significant role in most cases of chronic urticaria.

#### Acute urticaria

The most common causes of acute urticaria (with or without angioedema) are medications, foods, viral infections, stress, parasitic infections, insect venom, and contact allergens (e.g., latex) [9]. Medications known to commonly cause urticaria with or without angioedema include antibiotics (particularly beta lactams and sulfonamides), non-steroidal anti-inflammatory drugs (NSAIDs), acetylsalicylic acid (ASA), opiates and narcotics. The predominant foods that cause urticaria are milk, eggs, peanuts, tree nuts, fish, and shellfish. Acute urticaria should be differentiated from anaphylaxis which has similar triggers including food, medication, and insect stings; however, treatment approaches will be different. In approximately 50% of patients with acute urticaria, the cause is unknown and the condition is referred to as acute spontaneous urticaria (ASU) [1–3, 10]. Up to 36% of patients with ASU can progress to CSU [11].

#### Inducible urticaria

Inducible urticaria is triggered by a physical stimulus. The most common physical urticaria is dermatographism (also known as “skin writing”), in which lesions are created or “written” on the skin by stroking or scratching the skin. Pressure areas from clothing such as the waistline (i.e., after wearing tight-fitting pants) and the area of the ankles or calves that makes contact with the elastic band of socks are commonly affected [1–3, 10, 12]. Cholinergic urticaria is also common and results from a rise in basal body temperature that occurs following physical exertion or exposure to heat. Other physical stimuli which can trigger urticaria include exposure to cold (cold-induced urticaria), ultraviolet light (solar urticaria), water (aquagenic urticaria), vibration and exercise. The lesions produced by these physical stimuli are typically localized to the stimulated area and often resolve within 2 h. However, some patients may experience delayed-pressure urticaria which, as the name implies, comes on slowly (i.e., 30 min to 12 h) after pressure has been applied, and can last several hours or even days. Typical areas affected include the hands and feet, especially when constant pressure is applied to these areas during specific tasks or in certain occupations.

### Diagnosis

The diagnosis of urticaria, with or without angioedema, is based primarily on a thorough clinical history and physical examination. Based on the history and physical exam, diagnostic tests may also be considered to help confirm a diagnosis of acute, chronic or inducible urticaria.

#### History and physical examination

The history and physical examination should include detailed information regarding: the frequency, timing, duration and pattern of recurrence of lesions; the shape, size, site and distribution of lesions; potential triggers (e.g., food, medications, physical stimuli, infections, insect stings, stressful occurrences); response to previous therapies used; and a personal or family history of atopy [5–7]. Many conditions can easily be confused with urticaria, particularly urticarial vasculitis and systemic mastocytosis (see Table 1 for conditions that need to be considered in the differential diagnosis of urticaria). In urticarial vasculitis, the lesions are usually painful rather than pruritic, last longer than 48 h, and leave bruises or discoloration on the skin [1, 13]. Systemic mastocytosis (also called systemic mast cell disease) is a rare condition that involves the internal organs, in addition to the skin. In this disorder, atypical mast cells collect in various tissues that can affect the liver, spleen, lymph nodes, bone marrow and other organs [1, 10]. Initially, urticaria can be confused with erythema multiforme, and vice versa, but the latter develops quite differently with blistering, which does not occur in urticaria.

**Table 1:** Conditions to consider in the differential diagnosis of urticaria

Urticarial vasculitis	• Lesions are usually painful (rather than pruritic), last > 48 h, and leave discoloration on the skin
Systemic mastocytosis	•Rare condition that involves the internal organs (liver, spleen, lymph nodes, bone marrow), in addition to the skin
Atopic dermatitis	•Chronic, highly pruritic inflammatory skin disease •Clinical manifestations vary with age
Bullous pemphigoid	•Chronic, autoimmune, blistering skin disease
Erythema multiforme	•Acute, self-limited, skin condition •Considered to be a type IV hypersensitivity reaction to certain infections, medications, and other various triggers
Familial cold autoinflammatory syndrome	•Rare, inherited inflammatory disorder characterized by recurrent episodes of rash, fever/chills, joint pain, and other signs/symptoms of systemic inflammation triggered by exposure to cooling temperatures •Onset usually occurs during infancy and early childhood and persists throughout the patient’s life
Fixed drug eruptions	•Lesions occur from exposure to a particular medication and occur at the same site upon re-exposure to the offending medication •Lesions usually blister and leave residual pigmentation
Subacute cutaneous lupus erythematosus	•A non-scarring, photosensitive skin condition •May occur in patients with systemic lupus erythematosus (SLE) and Sjögren syndrome
Pruritic urticarial papules and plaques of pregnancy	•Benign skin condition that usually arises late in the third trimester of a first pregnancy
Muckle–Wells syndrome	•Rare genetic disease that causes hearing loss and recurrent hives •May lead to amyloidosis
Schnitzler’s syndrome with monoclonal IgG kappa gammopathy	•Rare disease characterized by chronic, non-pruritic hives, periodic fever, bone and joint pain, swollen lymph glands and an enlarged spleen and liver

#### Diagnostic tests

Skin prick tests (SPTs) and serum-specific IgE tests may help confirm a diagnosis of acute urticaria resulting from allergic or IgE-mediated (type I) reactions to common food allergens, latex hypersensitivity, stinging insect hypersensitivity and certain antibiotics. These tests are best performed by allergists with experience in interpreting test results in the appropriate clinical context.

Certain diagnostic tests and assessments can be helpful in the diagnosis and differential diagnosis of CSU, including: a complete blood count (CBC), and erythrocyte sedimentation rate (ESR) or C-reactive protein (CRP) as markers of inflammation [5–7]. The presence of thyroid autoantibodies supports the autoimmune process in CSU. If there are atypical features, a skin biopsy, assessment of serum tryptase and complement levels, and serum protein electrophoresis should be considered.

The autologous serum skin test (ASST) involves intradermal injection of the patient’s own serum (collected while the patient is symptomatic) into uninvolved skin. A positive wheal and flare reaction is considered indicative of circulating autoantibodies to the high-affinity IgE receptor or to IgE. However, it should be noted that the ASST is not widely used in clinical practice as it may not be specific for CSU. Since basophils are also involved in chronic urticaria, the basophil activation test (the quantification of basophil activation by flow cytometry) may be useful for screening for the autoimmune form of the disease. However, further confirmatory studies are needed before this test is widely accepted as a diagnostic tool [2].

Challenge testing, which reproduces exposure to a suspected stimulus in a supervised clinical environment, is often indicated to confirm a diagnosis of inducible urticaria. Cold-induced urticaria can usually be confirmed using the ice cube test (i.e., placing an ice cube in a sealed plastic bag over the forearm for 5–10 min). Dermatographism can be confirmed by lightly stroking the skin, or by using a standardized device such as a dermographometer. Aquagenic urticaria can be identified by immersion of a body part into warm water or through the application of warm compresses. Hot bath testing can help identify cholinergic urticaria, and the application of weight/pressure to the patient’s thigh or shoulder is helpful in the diagnosis of delayed-pressure urticaria [1, 2].

### Treatment

**Fig. 3:**
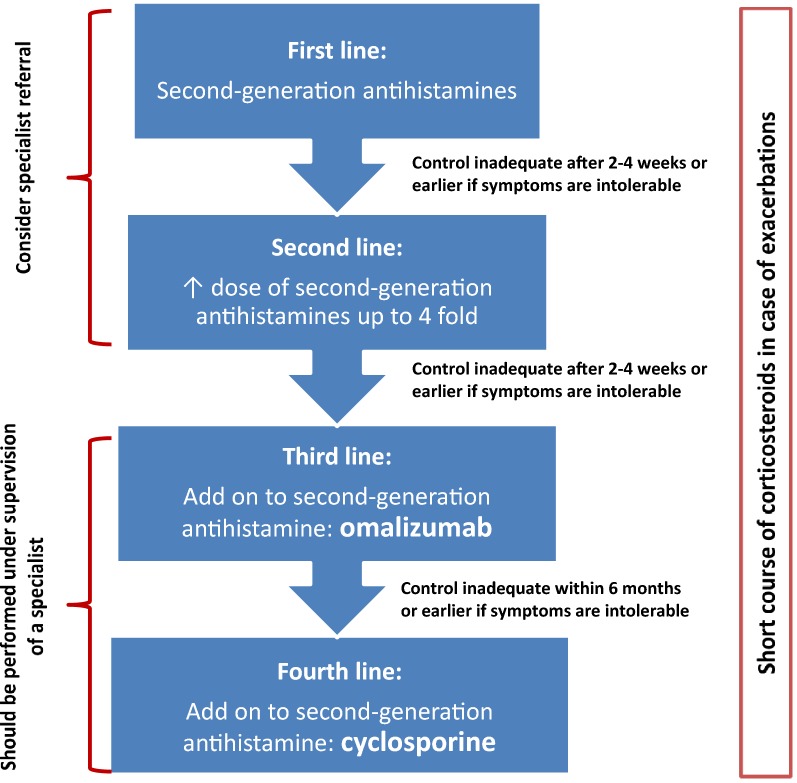
**Simplified stepwise algorithm for the treatment of urticaria** Adapted from Zuberbier et al. 2018 [15]

Strategies for the management of acute urticaria include avoidance measures, antihistamines and corticosteroids. For urticaria, antihistamines are the mainstay of therapy. Corticosteroids and various immunomodulatory/immunosuppressive therapies may also be used for more severe cases, or for those patients who experience a poor response to antihistamines (see Fig. 3).

#### Avoidance

For some patients with acute urticaria, a specific trigger can be identified (e.g., food, medication, insect venom, latex), and strict avoidance is recommended. Patients should be provided with clear, written instructions on appropriate avoidance strategies [2, 3]. These patients should be advised that exposure could not only lead to acute urticaria but also anaphylaxis.

For patients with CSU, NSAIDs, alcohol or opiates should be avoided as these can significantly exacerbate the condition. Food avoidance with elimination diets is not helpful for CSU.

#### Medications

Second-generation, non-sedating, non-impairing H1-receptor antihistamines (e.g., fexofenadine, desloratadine, loratadine, cetirizine, bilastine, rupatadine; see Table 2) are the mainstay of therapy for urticaria. These agents have been shown to be significantly more effective than placebo for the treatment of urticaria [5–7]. First-generation antihistamines should be avoided due to their anticholinergic effects and associated side effects (i.e., sedation and cognitive impairment). Histamine type 2 (H2)-receptor blockers (such as ranitidine and cimetidine) are not felt to be of benefit in the treatment of urticaria [14].

**Table 2:** Antihistamines commonly used and indicated for the treatment of urticaria

Second-generation H1-receptor antihistamines	Standard adult dose (mg daily)	4 times standard adult dose (mg daily)	Usual pediatric dose
Cetirizine (Reactine)	10–20	40	5–10 mL (1–2 teaspoons) daily (children’s formulation)
Desloratadine (Aerius)	5	20	2.5–5 mL (0.5–1.0 teaspoon) daily (children’s formulation)
Fexofenadine (Allegra)	120	480	Not currently indicated for children under 12 years of age
Loratadine (Claritin)	10	40	5–10 mL (1–2 teaspoons) daily (children’s formulation)
Bilastine (Blexten)	20	80	Not currently indicated for children under 12 years of age
Rupatadine (Rupall)	10	40	5–10 mL (1–2 teaspoons) daily (children’s formulation)

Acute spontaneous urticaria is usually treated with antihistamines at standard or higher doses. In severe cases, a short course of oral corticosteroids can be used.

Guidelines for the management of CSU have recently been updated (see Fig. 3) [15]. A standard dose of a second-generation antihistamine is the initial treatment. Antihistamine efficacy is often patient specific and, therefore, more than one antihistamine should be tried before assuming therapeutic failure with these agents. Also, antihistamines are most effective if taken daily, rather than on an as-needed basis [16]. If symptoms are controlled with standard antihistamine doses, it is reasonable to continue treatment for several weeks to months, occasionally stopping therapy for brief periods to determine whether the urticaria has spontaneously resolved. In patients who do not achieve adequate symptom control at standard doses in 2–4 weeks, it is common practice to increase the antihistamine dose beyond the usual recommended dose [17]. Canadian and European guidelines have recommended up to four times the usual recommended dose of antihistamines in patients whose symptoms persist with standard therapy [7].

For patients with CSU who have not responded to 4 times the standard dose of second-generation antihistamines after a 2- to 4-week trial, omalizumab, an anti-IgE humanized monoclonal antibody, as add-on therapy is now considered the third-line option (see Fig. 3). Randomized double-blind, placebo-controlled trials have demonstrated its efficacy and safety in patients with CSU refractory to H1-antihistamines [18, 19]. Both 150 and 300 mg of omalizumab injected subcutaneously every 4 weeks have been shown to be effective [19].

Cyclosporine is considered fourth-line therapy if there is no response to omalizumab within 6 months, or if the condition is intolerable. Small, double-blind, randomized controlled trials have found cyclosporine (3–5 mg/kg/day) to be effective in patients with chronic urticaria who do not adequately respond to antihistamines [20, 21]. During treatment with cyclosporine, H1-receptor antihistamines should be continued, and blood pressure, renal function and serum cyclosporine levels should be monitored regularly given the significant side effects associated with this form of therapy (e.g., hypertension, renal toxicity).

For some patients with severe urticaria, a brief course of oral corticosteroids (e.g., 0.3–0.5 mg/kg of prednisone for 10–14 days) is warranted. However, long-term corticosteroid therapy should be avoided given the well-known side effects associated with prolonged use and the increased likelihood of developing tolerance to these agents.

The leukotriene receptor antagonist (LTRA), montelukast, can be used as an add-on to second-line treatment in H1-antihistamine refractory CSU. However, some clinical trials have not found the addition of this LTRA to be of significant benefit [22].

Various immunosuppressive or immunomodulatory therapies may provide some benefit for patients with severe, chronic urticaria. Case reports and other small clinical trials have also found the following treatments to be effective for select patients with severe, refractory, chronic urticaria: sulfasalazine, dapsone, hydroxychloroquine, colchicine, mycophenolate mofetil, 5-aminosalicylic acid, and intravenous immunoglobulin G (IVIG) [7]. However, the efficacy of these agents in the treatment of chronic urticaria needs to be confirmed in large, randomized controlled trials. Also, it is important to note that these alternative treatments may have to be trialed given that anti-IgE therapy (omalizumab) is expensive and not available to all patients who fail high-dose, second-generation antihistamine therapy.

Quality of life can be significantly impacted in patients with CSU. The condition can have a negative impact on work, school, social activities, diet and sleep. Instruments to monitor QOL [e.g., Chronic Urticaria Quality of Life Questionnaire (CU-Q2oL)] and to quantify disease activity (the Urticaria Activity Score) have been developed. These tools can also assist in monitoring response to therapies [7].

## Angioedema

### Introduction

**Fig. 4:**
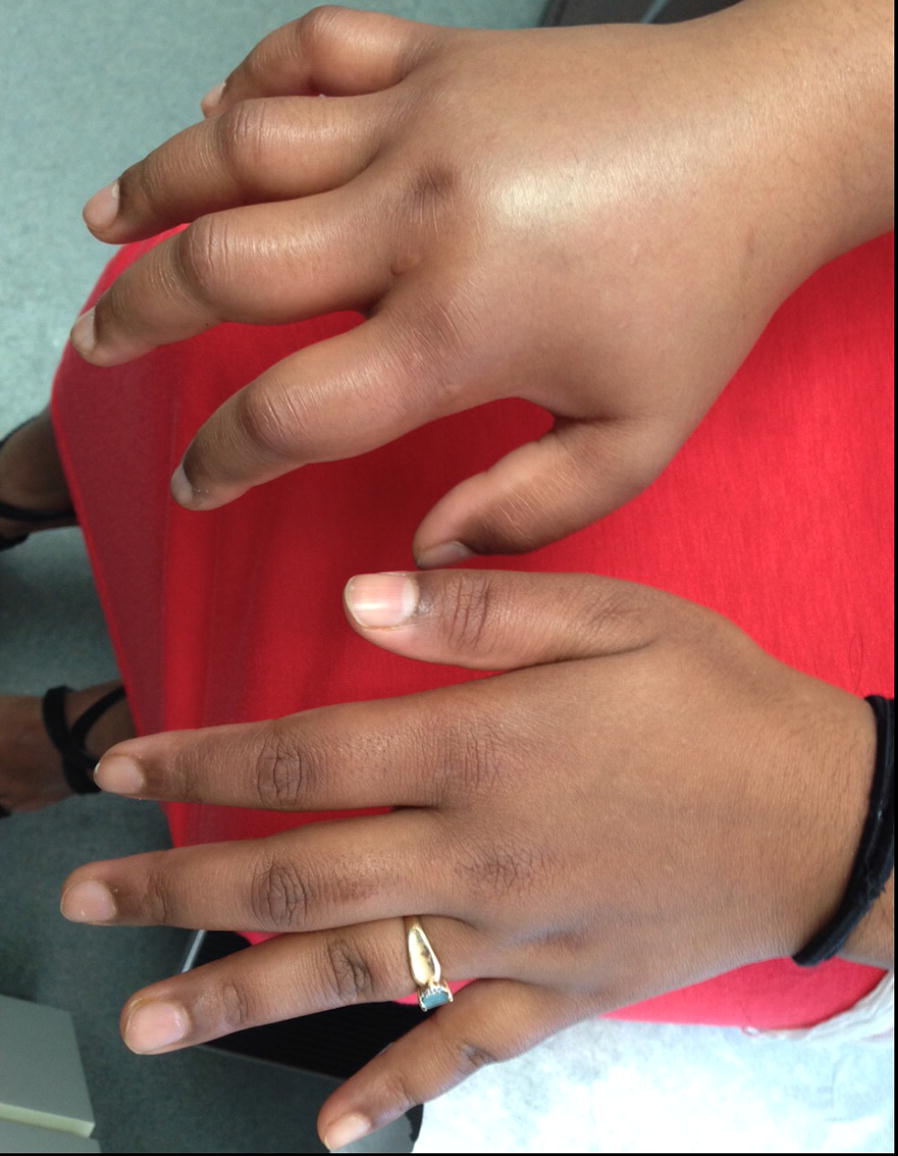
**Hereditary angioedema (HAE)**

**Fig. 5:**
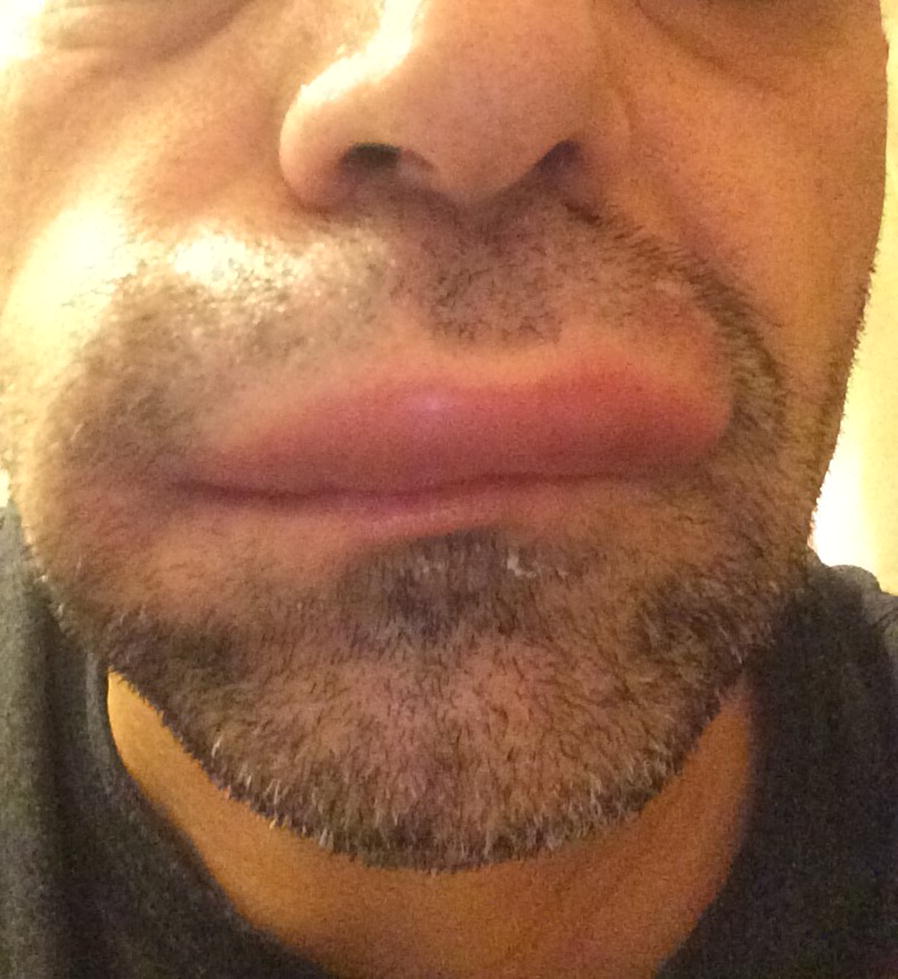
**Idiopathic angioedema**

Angioedema in the absence of urticaria (see Figs. 4 and 5) is much less common than urticaria either with or without angioedema. Its presence should alert the physician to alternative diagnoses, which could comprise life-threatening conditions including hereditary angioedema (HAE), acquired angioedema (AAE), or angioedema associated with angiotensin-converting enzyme (ACE) inhibitors.

Acute episodes of angioedema result from a release of vasoactive mediators that increase vascular permeability in the skin and submucosa, allowing for the vascular leakage of plasma and resultant edema; the majority of these attacks can be attributable to either histamine- or bradykinin-mediated mechanisms. Angioedema can be further classified as: histaminergic (through IgE independent or dependent mechanisms), idiopathic, hereditary (HAE type I [HAE-1], HAE type II [HAE-2] and HAE with normal C1 inhibitor [HAE-nC1-INH]), acquired (C1-inhibitor [C1-INH] deficiency from a secondary cause) and ACE inhibitor-induced. This section, although mentioning histamine-mediated angioedema, will focus on the classification, diagnosis and management of bradykinin-mediated angioedema.

#### Histamine-mediated angioedema

Histaminergic angioedema can be considered allergic, pseudoallergic or idiopathic. The approach to allergic and pseudoallergic (such as in the case of NSAIDs) angioedema is similar to that described earlier in the section on acute urticaria, understanding that these patients may not always have urticaria accompanying their angioedema.

In a significant number of patients, no identifiable cause is found for recurrent episodes of angioedema without urticaria; this is deemed as ‘idiopathic’ once alternate identifiable causes have been excluded. Idiopathic cases may occur in antibody-dependent and independent mechanisms, and typically respond to antihistamines and corticosteroids. Diagnosis is often made after ruling out other etiologies by assessing a patient’s response to antihistamine and corticosteroid therapy.

#### Bradykinin-mediated angioedema

In the case of HAE, AAE or ACE inhibitor-induced angioedema, the vasodilatory peptide, bradykinin, plays a key role in endothelial cell activation, with resultant tissue edema. Bradykinin is released from many cell types, and mechanisms that interfere in either its production—or as in the case of ACE inhibitors—its degradation, result in angioedema. This may occur through several mechanisms, namely the complement, coagulation and contact pathways which are essential in the regulation of bradykinin.

### Classification and etiology

Hereditary angioedema is a rare autosomal dominant genetic disorder resulting from an inherited deficiency or dysfunction of the C1 inhibitor (C1-INH; a plasma protease inhibitor that regulates several proinflammatory pathways). Two main types of HAE have been defined: type I [HAE-1] and type II [HAE-2]. HAE-1 is characterized by low C1-INH levels and function (85% of cases), while HAE-2 is associated with normal C1-INH levels, but low function (15% of cases). HAE with normal C1-INH level and function (HAE-nC1-INH) is inherited in an autosomal dominant manner and occurs mostly in females; however, some male patients have been reported. In some cases, HAE-nC1-INH may be associated with a gain of function mutation in factor XII, resulting in kinin overproduction [23, 24].

Acquired angioedema (AAE) is a rare C1-INH deficiency syndrome which is most commonly associated with B cell lymphoproliferative diseases (type 1 AAE). It may also be related to the presence of an autoantibody directed against the C1-INH molecule (type II AAE) [25].

Clinically, HAE and AAE are similar, and are characterized by recurrent episodes of angioedema, without urticaria or pruritus, which most often affect the skin or mucosal tissues of the gastrointestinal and upper respiratory tracts. Although generally benign conditions, laryngeal involvement can rapidly lead to fatal asphyxiation if left untreated. Age of onset and the presence of a familial history are distinguishing features of these conditions (see Table 3). HAE usually presents in late childhood or adolescence in otherwise healthy subjects, and a familial history is present in approximately 75% of cases (with the remaining 25% resulting from spontaneous mutation of the C1-INH gene). In contrast, AAE is not associated with a family history, and usually develops in older patients (fourth decade of life) with an underlying lymphoproliferative or autoimmune disease [25].

**Table 3:** Comparison of HAE and AAE [23, 33]

	Family history	Complement levels/laboratory findings		
		C4	C1-INH antigen	C1-INH function
HAE-1 HAE-2	Yes^a^	↓ ↓	↓ Normal or ↑	↓ ↓
HAE-nC1-INH				
FXII mutation	Yes	Normal	Normal	Normal
Unknown cause				
AAE	No	↓	Normal or ↓	↓

Adapted from Betschel et al. [23], Cicardi et al. [33]

^a^In approximately 25% of patients, no family history is identified; the disorder results from spontaneous mutation of the C1 inhibitor gene

Although the exact pathogenesis of attacks of HAE and AAE remains unclear, excess production of the potent vasodilatory peptide, bradykinin (which is regulated by the C1-INH), appears to play an important role [26]. It is important to note that histamine and other mast cell mediators that are typical of urticaria and associated angioedema are not directly involved in HAE and AAE, which explains patient lack of response to antihistamines and corticosteroids, and distinguishes these forms of isolated angioedema from that associated with urticaria.

Angiotensin-converting enzyme inhibitor-induced angioedema is one of the most common causes for emergency treatment of angioedema [27]. It occurs in approximately 0.1–6% of individuals using ACE inhibitors, and tends to occur more commonly in ACE inhibitor users who are female, smokers, or of African-American descent. Like HAE and AAE, ACE inhibitor–induced angioedema is bradykinin-mediated. Most cases of angioedema occur in the first week after starting ACE inhibitor therapy. However, up to one-third of cases occur months to years after initiating the medication [28]. ACE inhibitor-induced angioedema can be life-threatening when it involves the upper airway. Therefore, ACE inhibitors should be discontinued in all individuals with angioedema, and are absolutely contraindicated in patients with either HAE or AAE. Episodes of angioedema may occur up to 1 month (or sometimes more) after discontinuing the ACE inhibitor.

### Diagnosis

The diagnosis of HAE and AAE is based upon a suggestive clinical history, and there is significant overlap in their clinical presentation. The angioedema may present in the face, extremities, abdomen and other organ systems, with the concern of laryngeal edema and asphyxiation. The most common presentation is that of non-emergent angioedema resulting in impairment in QOL with discomfort, immobility and disfigurement, and the inability to attend work or school [29, 30].

Abdominal attacks occur in up to 93% of patients with HAE [31]. These attacks can present with mild to severe spasmodic pain, and may be associated with gastrointestinal upset and even intestinal obstruction; hypovolemic shock may result from the extravasation of fluids. The attacks can often be confused with appendicitis or cholecystitis, resulting in unnecessary surgical interventions, and even psychiatric referrals.

Laryngeal attacks with respiratory impairment and the risk of asphyxiation is the most feared complication of these attacks, as these patients may require intubation and even tracheotomies. It is estimated that up to 50% of HAE patients will experience at least 1 laryngeal episode within their lifetime [32].

Complement studies that should be ordered for patients with suspected HAE and AAE include: levels of C4 (the natural substrate for C1), C1q, C1-INH antigen, and function of C1-INH [23]. Ideally, these studies should be performed when the patient is not receiving treatment since the use of therapeutic interventions for AAE or HAE can alter laboratory results. Patients with AAE should be evaluated for an underlying B-cell lymphoproliferative disorder at the time of diagnosis [23].

In HAE-1, C1-INH antigenic and functional levels are low (< 50% of the lower limit of normal). In HAE-2, C1-INH functional levels are low, but antigenic levels are normal or elevated (see Table 3) [23, 33]. In HAE-nC1-INH, C4, C1-INH level and function, and C1q are all normal. In most patients with AAE, C4, C1q, and C1-INH function levels are low (< 50% of the lower limit of normal), and C1-INH antigenic levels are low or normal.

Angiotensin-converting enzyme inhibitor-induced angioedema should be suspected in any patient who is on an ACE inhibitor and develops angioedema without urticaria. Although complement studies are normal in ACE inhibitor-induced angioedema, C1-INH levels should be measured in these patients as the initiation of an ACE inhibitor could unmask HAE or AAE.

### Treatment

Most of the evidence for managing bradykinin-mediated angioedema is based on clinical trials in HAE. The management of HAE can be divided into the following approaches: treatment of acute attacks, short-term prophylaxis (STP), and long-term prophylaxis (LTP). The aim of treatment of acute attacks, also referred to as ‘on demand therapy’, is to minimize their severity, including potentially fatal upper airway edema and associated impairment of QOL. STP refers to treatment meant to minimize the risk of attacks when avoidance of potential and known triggers is not possible. LTP refers to ongoing treatment of HAE aimed at minimizing the overall number, frequency and/or severity of attacks [23].

#### Treatment of acute attacks

First-line therapies for the treatment of acute attacks of HAE and AAE include: C1-INH replacement therapy, icatibant and ecallantide [23]. The goal of acute treatment is to treat early to minimize the impact angioedema has on morbidity and mortality. All patients should have access to an acute therapy that facilitates early treatment and that is best suited to their individual needs. Since these patients may present to healthcare providers who may not be familiar with their condition, it is advisable that patients carry a wallet card or similar tool which outlines their condition and their specific treatments.

Treatment with C1-INH replaces the deficient protein in patients with HAE-1 and HAE-2. Berinert, a plasma-derived C1-INH, is the only approved C1-INH replacement product in Canada to treat acute attacks. It is administered as an intravenous rapid push at a dose of 20 units/kg. Ruconest, a recombinant form of C1-INH is available in other countries, but not approved for use in Canada. Although C1-INH replacement is used to treat AAE, some patients may become non-responsive to this treatment over time; in these patients, the use of ecallantide and icatibant (described below) could be considered.

Icatibant is a bradykinin receptor blocker which is approved in Canada for the treatment of acute attacks of HAE-1 and -2. The usual recommended dose for adults is 30 mg subcutaneously. Although evidence suggests that icatibant is also effective for the treatment of ACE inhibitor-induced angioedema [34], this is not an approved indication. The most common side effects of icatibant are mild and transient injection-site reactions. Other, less common side effects include: nausea, gastrointestinal upset, asthenia, dizziness, and headache [23].

Ecallantide, which has not been approved in Canada, is an inhibitor of plasma kallikrein (the enzyme that releases bradykinin, the primary mediator of angioedema). Although the side effects of ecallantide are generally mild (i.e., injection-site reactions, headache, nausea, fatigue, diarrhea), this therapy has been associated with rare instances of allergic reactions and anaphylaxis. Therefore, it should only be administered by a clinician in a medical setting equipped to manage anaphylaxis and severe angioedema [23].

#### Short-term prophylactic treatment

Short-term prophylaxis (STP) refers to the practice of treating patients to reduce the risk of associated and consequent morbidity and mortality during a period of time when there may be an increased risk of having an attack of angioedema [23]. Triggers for attacks include physical trauma, such as that which may occur during medical and dental procedures. Attacks can occur anywhere from hours to several days after a procedure. Upper airway manipulation (e.g., dental surgery and intubation) is considered particularly high risk due to its association with upper airway swelling. It is also suspected that other causes, such as emotional stressors, can precipitate attacks. Individual patients may also be aware of specific triggers that have been known to trigger their attacks.

No specific treatments are approved in Canada for STP. However, C1-INH replacement is approved in Europe. Cinryze is licensed to be given within 24 h of an anticipated procedure at a dose of 1000 units, while Berinert is licensed for use within 6 h of a procedure (also at a dose of 1000 units).

Attenuated androgens may be considered for STP when surgery-related risks are considered low and other HAE-specific acute treatments are not immediately available. If androgens are selected for STP, danazol (2.5–10 mg/kg/day, maximum 600 mg/day) can be considered and should be initiated 5 days before the anticipated procedure or trigger and continued 2–3 days after the anticipated trigger [23].

#### Long-term prophylactic treatment

There are no specific criteria for determining when LTP should be initiated in patients with HAE or AAE. There is general agreement that suitable patients for LTP include those who experience 2 or more attacks per month, who have had recurrent laryngeal attacks, or in whom treatment for acute episodes is not sufficiently effective. However, even in these patients, individual factors need to be considered before determining appropriateness for LTP. Also, since these patients still have acute attacks, they must have access to acute treatment despite being on prophylaxis [23].

Factors triggering acute attacks of AAE and HAE vary but often include: mild trauma to the face (particularly dental trauma), stress/anxiety, *H. pylori* infection, menstruation, and the use of estrogen-containing medications (e.g., hormone replacement therapy and contraceptives) and ACE inhibitors. Whenever possible, these triggers should be avoided.

The best evidence for LTP for HAE-1 and -2 is with plasma-derived C1-INH replacement therapy [23]. Cinryze, a plasma-derived C1-INH, is approved for LTP in Canada at a dose of 1000 U intravenously every 3–4 days. This treatment can usually be administered at home by the patient or caregiver. Since C1-INH replacement therapy is a blood product, annual recipient hemovigilance and vein-to-vein tracking are essential.

Attenuated androgens, such as danazol, increase C4 and C1-INH levels and may be used for LTP in HAE and AAE [23]. Although generally well-tolerated by most patients, the adverse effects of long-term androgen administration may include: virilization, abnormalities in serum transaminases, menstrual irregularities, hair growth, decreased libido, weight gain, vasomotor symptoms, lipid abnormalities, and depression. Therefore, the lowest effective dose should be utilized (maximum long-term recommended dose for danazol is 200 mg daily), and the patient’s CBC, liver enzymes and lipid profile should be monitored regularly (e.g., every 6 months) while on therapy. Contraindications to androgen therapy include: pregnancy, lactation, cancer, hepatitis, and childhood.

There is less evidence to support the use of the antifibrinolytic agent, tranexamic acid, for the prophylactic treatment of HAE and AAE [23]. Tranexamic acid is well-tolerated and is generally preferred for LTP in pregnant women, children, and patients who do not tolerate androgens. The most common side effect is dyspepsia, which can be reduced by taking the drug with food.

## Conclusions

Urticaria is a common disorder that often presents with angioedema. It is generally classified as acute (lesions occurring for < 6 weeks), chronic (lesions occurring for > 6 weeks) and inducible (lesions result from a physical stimulus). The disorder can usually be diagnosed on the basis of clinical presentation and history; however, additional investigations may be helpful for confirming the diagnosis. Second-generation, non-sedating, non-impairing H1-receptor antihistamines represent the mainstay of therapy for both acute and chronic urticaria, and up-dosing of these agents can result in better control for some individuals. First-generation antihistamines should be avoided due to their sedating, impairing and anti-cholinergic side effects. For severe, chronic urticaria, omalizumab and cyclosporine are considered third- and fourth-line therapies, respectively. Short courses of oral corticosteroids can provide temporary benefit, but long-term use is discouraged.

Angioedema can occur in the absence of urticaria. The more common causes are ACE inhibitor-induced angioedema and idiopathic angioedema. Rare, but life-threatening, causes are HAE or AAE. The work-up and management of HAE and AAE vary considerably from that of angioedema associated with urticaria. Although the angioedema associated with these disorders is often self-limited, laryngeal involvement can lead to fatal asphyxiation. Patients with these disorders demonstrate characteristic abnormalities in certain complement levels and, therefore, diagnostic testing of patients with suspected HAE or AAE should include assessment of C4 and C1q levels, and C1-INH function and antigenic levels. HAE should be considered in patients with an early age of onset and a family history of the disorder; in patients with AAE, there is no family history and age of onset is usually later. All patients with HAE and AAE must have access to an effective acute treatment, and measures should be taken to minimize the time to administration of acute therapy. STP must be considered for all patients with HAE or AAE during times when the risk of angioedema is increased. LTP should be considered in patients with HAE and AAE when their attack frequency and severity are not adequately controlled with acute therapy alone.

## Key take-home messages

- Urticaria is a common disorder characterized by recurrent, pruritic (itchy) lesions with pale centers (wheals) that usually subside within 48 h; it is often associated with angioedema.
- Mast cells are the primary effector cells in urticaria.
- Urticaria is classified as acute (lesions for < 6 weeks), chronic (lesions for > 6 weeks), or inducible.
- The diagnosis of urticaria, with or without angioedema, is based primarily on a thorough clinical history; however, diagnostic tests may be helpful in some instances.
- Second-generation, non-sedating H1-receptor antihistamines are the mainstay of therapy for urticaria. Omalizumab and cyclosporine can be used for more severe, chronic cases.
- Angioedema can occur in the absence of urticaria, with ACE inhibitor-induced and idiopathic angioedema being the most common causes.
- ACE inhibitors should be discontinued in any individual who presents with angioedema as this condition is associated with life-threatening upper airway angioedema.
- Idiopathic angioedema responds well to prophylactic antihistamines; however, oral corticosteroids may be required in some cases.
- HAE and AAE are rare disorders also characterized by angioedema in the absence of urticaria; they result from a deficiency or dysfunction of the C1-INH (a plasma protease inhibitor that regulates several proinflammatory pathways), and are associated with life-threatening upper airway swelling.
- The diagnosis of HAE and AAE should include the assessment of C4, C1q, and C1-INH function and antigenic levels.
- The management of these disorders involves an approach to acute treatment, short-term and long-term prophylaxis that is evidence-based and follows national and international guideline recommendations.

## Abbreviations

ACE: angiotensin-converting enzyme
HAE: hereditary angioedema
HAE-1: hereditary angioedema type I
HAE-2: hereditary angioedema type II
AAE: acquired angioedema
Ig: immunoglobulin
QOL: quality of life
CSU: chronic spontaneous urticaria
SLE: systemic lupus erythematosus
IVIG: intravenous immunoglobulin G
NSAIDs: non-steroidal anti-inflammatory drugs
ASA: acetylsalicylic acid
ASU: acute spontaneous urticaria
SPTs: skin prick tests
CBC: complete blood count
ESR: erythrocyte sedimentation rate
CRP: C-reactive protein
ASST: autologous serum skin test
CU-Q2oL: Chronic Urticaria Quality of Life Questionnaire
HAE-nC1-INH: hereditary angioedema with normal C1 inhibitor
C1-INH: C1-inhibitor
STP: short-term prophylaxis
LTP: long-term prophylaxis
LTRA: leukotriene receptor antagonist
H1: histamine type 1
H2: histamine type 2
